# Anti-Inflammatory and Pro-Inflammatory Adipokine Profiles in Children on Vegetarian and Omnivorous Diets

**DOI:** 10.3390/nu10091241

**Published:** 2018-09-06

**Authors:** Jadwiga Ambroszkiewicz, Magdalena Chełchowska, Grażyna Rowicka, Witold Klemarczyk, Małgorzata Strucińska, Joanna Gajewska

**Affiliations:** 1 Department of Screening and Metabolic Diagnostics, Institute of Mother and Child, Kasprzaka 17A, 01-211 Warsaw, Poland; magdalena.chelchowska@imid.med.pl (M.C.); joanna.gajewska@imid.med.pl (J.G.); 2 Department of Nutrition, Institute of Mother and Child, Kasprzaka 17A, 01-211 Warsaw, Poland; grazyna.rowicka@imid.med.pl (G.R.); witold.klemarczyk@imid.med.pl (W.K.); malgorzata.strucinska@imid.med.pl (M.S.)

**Keywords:** anti-inflammatory adipokines, pro-inflammatory adipokines, vegetarian diet, body composition, children

## Abstract

Adipose tissue is a highly active endocrine organ that secrets many pro-inflammatory as well anti-inflammatory adipokines. The aim of the study was to assess serum adipokine profile in prepubertal vegetarian and omnivorous children. Sixty-two children on a vegetarian diet and fifty-five children on an omnivorous diet, aged 5 to 10 years, were studied. Dietary assessment was performed using a nutritional software program. Body composition was measured by dual-energy X-ray absorptiometry. Serum concentrations of adipokines: leptin, soluble leptin receptor (sOB-R), adiponectin (total and high molecular weight), resistin, visfatin, vaspin, and omentin were determined by immunoenzymatic assays. Both studied groups of children were comparable in terms of age, weight, height, body mass index, and body composition. Vegetarians had a lower (*p* = 0.017) leptin/sOB-R ratio and lower serum concentrations of resistin (*p* = 0.051), compared with omnivores. Average levels of other adipokines did not differ between both groups of children. However, we observed significantly higher ratios of anti-inflammatory to pro-inflammatory adipokines: adiponectin/leptin 0.70 (0.37–0.93) vs 0.39 (0.28–0.74), *p* = 0.005, and omentin/leptin 0.40 (0.23–0.83) vs. 0.33 (0.15–0.48), *p* = 0.011 in vegetarians compared with omnivores. A well-planned vegetarian diet might beneficially affect the adipokine profile and inflammatory status expressed by the ratios of anti-inflammatory to pro-inflammatory adipokines in prepubertal children.

## 1. Introduction

Recent studies have focused on the effects of diet patterns on health outcomes; however, little is known about links between diet type and the mechanisms regulating metabolic processes [1,2,3]. Adipokines, secreted by adipose tissue, are involved in an autocrine and paracrine manner in the regulation of energy expenditure, insulin sensitivity, glucose and lipid metabolism, endothelial function, and inflammation [4,5,6]. Their functions are pluripotent, numerous adipokines have been associated with inflammatory responses in different ways; some of them are related to pro- and others to anti-inflammatory conditions.

Among adipokines, leptin, resistin, and visfatin were described as markers that are positively related with body weight, fat mass, insulin resistance, and pro-inflammatory properties. Leptin, the first adipokine discovered, influences many metabolic processes, as well as immunological and inflammatory status [7,8]. It acts through its receptors; one of these is the soluble leptin receptor (sOB-R), which is reported to be the main leptin binding protein. The ratio of leptin to sOB-R corresponds with leptin action, determines leptin resistance, and is known to be a better indicator of metabolic complications than leptin levels alone [9]. Another important adipokine, resistin, is synthesized not only in adipose tissue, but also in macrophages. Therefore, resistin has pro-inflammatory properties and its increased levels indicate the development of insulin resistance, diabetes, obesity, and cardiovascular disease [10]. Visfatin, produced mainly by visceral adipose tissue, is a multifunctional protein, acting as a cytokine, hormone, and enzyme. Its form, NAMPT (nicotinamide phosphoribosyltransferase) visfatin has insulin-mimetic and adipogenic properties [11,12].

Opposite to the pro-inflammatory cytokines, adipose tissue can secrete a series of anti-inflammatory adipokines, including adiponectin and omentin, which play crucial protective roles in inflammation states. Adiponectin acts by binding to its receptors, and it possesses anti-inflammatory, anti-atherogenic, and insulin-sensitizing properties. It circulates not only as total adiponectin, but in three forms that differ in multimerization and biological activity. Among these forms, high-molecular weight multimers (HMW) appear to have the highest bioactivity, and the HMW/total adiponectin ratio is a better determinant of metabolic status than total adiponectin alone [13,14]. Omentin, secreted by the stroma cells of adipose tissue, has anti-inflammatory properties via the activation of endothelial nitric oxide synthesis, blood pressure regulation, and by directly influencing blood vessel relaxation [15,16,17,18,19]. Also, vaspin, a member of the serine protease inhibitor family, plays an important role of protecting against inflammation and atherosclerosis [20]. It acts through inhibiting the nuclear factor-kappa B (NF-kB), protecting vascular endothelial cells [21,22].

Several studies have shown the beneficial effects of plant-based diets on cardiovascular risk, inflammation states, and adipokine levels, but the underlying mechanisms for this influence are not fully understood [2,3,23,24]. The authors reported that the Mediterranean diet was associated with increased serum adiponectin concentrations and decreased levels of leptin and inflammatory markers (C-reactive protein, interleukin 6) [25,26,27]. It is necessary to clarify whether changes in adipokine levels are affected by dietary patterns independent of body mass index changes. Some researchers confirmed that the effect of diet on plasma adiponectin is independent of body weight status [1,28].

The vegetarian eating style is becoming popular in many countries, and an increasing number of families, including children and adolescents, choose vegetarian diets [29,30,31,32]. According to the statements of the Dietetic Association, a well-planned vegetarian diet is adequate for all stages of the life cycle, including childhood [33,34]. Also, several studies have shown the beneficial effects of the vegetarian diet on the decreased risk of cardiovascular diseases, hypertension, type 2 diabetes, and dyslipidemia [35,36,37]. There is little data about the impact of the vegetarian diet on the adipokine profile. The authors observed lower serum leptin, and higher or unchanged adiponectin concentrations in vegetarians [36,38,39]. No data exists regarding the serum concentrations of novel adipokines, such as visfatin, vaspin, and omentin, in subjects consuming a vegetarian diet.

The aim of this study was: (1) to evaluate serum concentrations of selected adipokines; (2) to analyze correlations between adipokine levels and anthropometric as well nutrition parameters; and (3) to compare the ratios of anti-inflammatory to pro-inflammatory adipokines in prepubertal children on vegetarian and omnivorous diets.

## 2. Methods

### 2.1. Subjects

The protocol of this study was in accordance with the Helsinki Declaration of Principles, and approved by the Ethics Committee of the Institute of the Mother and Child (decision number 12/2017). Written informed consent was obtained from all children’s parents. Children were recruited between February 2017 and April 2018 from a group of consecutive patients attending the Department of Nutrition at the Institute of Mother and Child in Warsaw.

We examined 62 prepubertal children (54.8% male, age range 5–10 years) following a lacto-ovo-vegetarian diet, which included dairy and egg products. The inclusion criteria regarding vegetarians were: being on a lacto-ovo-vegetarian diet from birth, in the prepubertal period, generally healthy (without development and nutrition disorders, normal-weight with a body mass index (BMI) *z*-score between −1 + 1). The exclusion criteria were: not in the prepubertal period, history of low birth weight, gastrointestinal diseases accompanied by malabsorption, history of acute and chronic infection, and drug consumption, except of standard vitamin D supplementation. In this study, we recruited the maximum possible number of prepubertal vegetarian children who consumed a lacto-ovo-vegetarian diet from birth.

The control group included 55 healthy children (49.1% male, age range 5–10 years) following a traditional omnivorous diet, which consisted of consuming meat, poultry, and fish.

Pubertal stage was assessed according to the Tanner’s criteria. The studied children (vegetarians and omnivores) attained the World Health Organisation (WHO)recommendation regarding physical activity and accumulated about 60–90 min/day of MVPA (moderate-to-vigorous physical activity) and approximately 30 min per day of VPA (vigorous physical activity). VPA included activities after school twice a week for 1 or 2 h. The level of physical activity was similar in both studied groups of children.

### 2.2. Dietary Assessment

Dietary assessment in the studied children had been described in detail previously [40]. The parents of studied children (advised by a nutritionist) were asked to prepare a food diary for their children. Three consecutive days (two weekdays and one weekend day) were selected, and data were analyzed by a nutritional software program Dieta5® (National Food and Nutrition Institute, Warsaw, Poland). The average daily energy intake, dietary protein, fat, carbohydrates, and fiber intakes was assessed in the studied children, and compared with the current recommendations for Polish children [41].

### 2.3. Anthropometric Parameters

Physical examination included height and weight measurements, and were performed in all children. Body mass index (BMI) was calculated as the body weight (kg) divided by height squared (m^2^). Fat mass, lean mass, and fat free mass were measured by dual-energy X-ray absorptiometry (DXA) using Lunar Prodigy (General Electric Healthcare, Madison, WI, USA). Fat mass index (FMI) and lean mass index (LMI) were calculated as the fat mass or lean mass (kg) divided by the height squared (m^2^), respectively.

### 2.4. Biochemical Measurements

Blood samples were obtained after an overnight fast (between 8:00–10:00 a.m.), and serum samples were frozen at −20 °C until analysis (no longer than after two months). Concentrations of serum adipokines were determined by a commercial enzyme-linked immunosorbent assay (ELISA) kit, according to the manufacturer’s instructions. Serum leptin and soluble leptin receptor (sOB-R) concentrations were determined using ELISA kits from DRG Instruments GmbH (Marburg, Germany), with intra-assay variation coefficients (CV) ranging between 4.2–7.3%, and inter-assay CV ranging between 3.7–9.1% for leptin, and intra-assay CV 7.10–7.23% and inter-assay CV 6.21–9.81% for sOB-R. Serum total and HMW adiponectin levels were measured using a kit from ALPCO (Salem, MA, USA), with intra-assay CV 5.3–5.4% for total adiponectin and 3.3–5.0% for HMW adiponectin, and inter-assay CV 5.0% for total adiponectin and 5.7% for HMW adiponectin, respectively. The resistin level was determined using a kit from DRG Instruments GmbH (Marburg, Germany), with intra-assay CV 5.2–6.6% and inter-assay CV 7.0–8.1%. The concentrations of vaspin and visfatin were determined using a Vaspin (human) ELISA kit and Nampt (Visfatin/pre-B cell colony-enhancing factor (PBEF) (human) kits from Adipogen Life Science (Liestal, Switzerland). The intra-assay and inter-assays were 1.31–3.85% and 3.27–9.06% for vaspin, and 2.31–9.11% and 4.66–7.24% for visfatin, respectively. Serum omentin was measured using a kit from SunRed Biotechnology (Shanghai, China), with a within-assay variability of less than 10% and a between-assay variability of less than 12%.

### 2.5. Statistical Analysis

All statistical analyses were carried out using IBM-SPSS software, version 23.0 (SPSS INC., Chicago, IL, USA). The Kolmogorov-Smirnov test was used to test the normality of the distribution of the variables. For compare categorical data the chi-squared test was used. Baseline characteristics were presented as mean ± standard deviation (SD) for the variables with normal distribution, or as median values and interquartile ranges (IQRs) for the variables that were non-normally distributed. The ratios of anti- to pro-inflammatory cytokines: adiponectin/leptin, adiponectin/resistin, omentin/leptin, omentin/resistin, vaspin/leptin, and vaspin/resistin were calculated. The differences between groups regarding anthropometrical, nutritional, and biochemical parameters were evaluated using the Student’s *t*-test for normally distributed variables, and the Mann-Whitney *U* test for non-parametric variables. A univariate correlation analysis was performed using the Pearson or Spearman test, as appropriate. A *p* value of <0.05 was considered to be statistically significant.

## 3. Results

Both groups of children were similar in terms of age, body weight, height, BMI, and lean and fat mass (Table 1). The mean values of LMI were similar in both studied groups of children, but FMI was significantly (*p* = 0.044) lower in the vegetarian than in the omnivorous children. Analyzing the children’s diets, we found that the average daily energy intake in both groups of children was within recommendations, whereas the proportions of macronutrient intakes in vegetarians and omnivores were different. Compared with omnivores, vegetarians had a similar percentage of energy from fat (*p* = 0.286), a significantly lower percentage of energy from protein (*p* < 0.001), and a higher percentage of energy from carbohydrates (*p* = 0.002). As expected, in the vegetarian diet, the daily intake of plant protein was significantly (*p* = 0.024) higher, and the intake of animal protein was significantly (*p* < 0.001) lower than in the omnivorous diet. Additionally, the dietary intake of fiber was higher (*p* = 0.011) in vegetarians compared with omnivores.

Among adipokine levels, we observed a significantly (*p* = 0.017) lower ratio of leptin to soluble leptin receptors, and the resistin concentration was on the border of statistical significance (*p* = 0.051) in vegetarians compared with omnivores (Table 2). Serum concentrations of other adipokines, such as adiponectin, visfatin, omentin, and vaspin, did not differ significantly between both groups of children; however, there was a slight increase in the median value of circulating visfatin and omentin.

When analyzing the correlations, we found a significant positive association between resistin and visfatin concentrations (*r* = 0.485, *p* < 0.001), and between resistin and the HMW/adiponectin ratio (*r* = 0.327, *p* = 0.009) in the group of vegetarian children, and between vaspin level and the leptin/sOB-R ratio (*r* = 0.287, *p* = 0.045) in the omnivorous group. We did not observe associations between other serum adipokine levels in any of the studied groups of children (data not shown). Regarding significant correlations between adipokines and anthropometric parameters, we noted that in vegetarians, the ratio of leptin/sOB-R was positively correlated with body weight (*r* = 0.371, *p* = 0.003), height (*r* = 0.400, *p* = 0.001), fat mass (*r* = 0.475, *p* = 0.000), fat/lean mass ratio (*r* = 0.346, *p* = 0.006), and FMI (*r* = 0.379, *p* = 0.002), and it was negatively correlated with LMI (*r* = −0.291, *p* = 0.022) (Table 3). Additionally, the HMW/total adiponectin ratio was negatively correlated with BMI (*r* = −0.379, *p* = 0.002) and FMI (*r* = −0.263, *p* = 0.039). Also, resistin was negatively correlated with fat mass (*r* = −0.305, *p* = 0.016), fat/lean mass (*r* = −0.303, *p* = 0.017), and FMI (*r* = −0.321, *p* = 0.011). In contrast, we did not observe correlations between adipokine concentrations and anthropometric data in the omnivorous group, except for a relation between the leptin/sOB-R ratio and weight (*r* = 0.292, *p* = 0.031), and between vaspin and LMI (*r* = 0.366, *p* = 0.016).

In the studied groups of children, we observed differences in the ratios of anti-inflammatory to pro-inflammatory adipokines (Table 4). Vegetarian children had a significantly higher adiponectin/leptin ratio: 0.70 (0.37–0.93) vs. 0.39 (0.28–0.74), (*p* = 0.005), and a higher omentin/leptin ratio: 0.40 (0.23–0.83) vs. 0.33 (0.15-0.48) (*p* = 0.011). We did not find significant differences in the ratios of adiponectin/resistin, omentin/resistin, vaspin/leptin, and vaspin/resistin between the studied groups.

We also analyzed correlations between the adipokine ratios and nutritional parameters (Table 5). We found that in vegetarians, the percentage of energy from protein was associated with the vaspin/resistin ratio (*r* = −0.294, *p* = 0.033), dietary fiber with the adiponectin/resistin ratio (*r* = −0.295, *p* = 0.032) and plant protein intake tended to be associated with the omentin/leptin ratio (*r* = 0.258, *p* = 0.062). In omnivores we observed that the percentage of energy from protein was negatively correlated with the adiponectin/leptin ratio (*r* = −0.335, *p* = 0.023), and the omentin/leptin ratio (*r* = −0.341, *p* = 0.032), and positively correlated with the vaspin/resistin ratio (*r* = 0.328, *p* = 0.039).

## 4. Discussion

Adipokines secreted by adipose tissue may act both locally and peripherally, regulating a large number of physiological processes. The dysregulation of adipokine synthesis can alter homeostasis and lead to pathological conditions [8]. The majority of research has focused on obesity and its impact on the adipokine profile, although a few papers consider the adipokine pattern in an undernutrition state, such as anorexia nervosa [42,43]. Analyzing adipokine status, Mantovani et al. [44] suggested that leptin, resistin, or adiponectin might be early markers of changing from lean to obesity, even before the occurrence of metabolic alterations. Generally, the researchers concluded that changes in nutritional status, as well in body weight and fat content, may influence the hormonal activity of adipose tissue, with special attention being paid to the balance between adipokines with pro-inflammatory and anti-inflammatory activities.

It is widely known that consuming a properly composed diet has been associated with better health status, due to the protective effect against various chronic diseases [37,45]. Evidence regarding the role of diet quality on glucose and lipid metabolism and inflammatory markers has been established in multiple observational studies [45,46,47,48,49]. The authors reported that a plant-based pattern and the Mediterranean diet (characterized by a high consumption of vegetables, fruits, legumes, olive oil, and low consumption of red meats) are associated with favorable health status and less inflammation [25,27]. Additionally, they observed that this kind of diet was associated with higher levels of adiponectin, lower levels of leptin, and unchanged levels of resistin and vaspin.

There is a paucity of data on the effects of the vegetarian diet on serum adipokine status [36,39]. We studied prepubertal children and found a different adipokine profile with a significantly lower leptin/soluble leptin receptor ratio, and resistin concentrations in vegetarians compared with omnivores. The changes in other adipokine levels were not statistically significant between the groups, but we noticed slightly higher serum omentin and visfatin concentrations in the vegetarian group. It is worth noting that we determined visfatin as nicotinamide phosphoribosyltransferase (NAMPT)—an enzyme form that plays a role in insulin secretion in pancreatic beta-cells, and in the delayed neutrophil apoptosis of sepsis [50,51]. However, visfatin is an adipokine that exerts pleomorphic effects, and it is not excluded that other forms of visfatin might have different properties and associations with anthropometric and nutritional parameters [52].

Significant positive correlations between the leptin/sOB-R ratio and anthropometric parameters (body weight, height, fat mass), between resistin and fat, and negative correlations between the HMW/total adiponectin and fat observed in our vegetarians (but not in omnivores) suggest exact association between adipokines and body composition in this group of children. We think that conditions with lower leptin and resistin levels, coexisting with lower fat mass and FMI in vegetarians, are favorable for health.

The interplay between adipokines of opposite effects, may determine their better metabolic profile regarding the inflammatory state. This is why we presented the ratios of anti- to pro-inflammatory adipokines reflecting an inflammatory balance that is better than the measurement of individual adiponectins. It is known that adiponectin and omentin have anti-inflammatory effects that contribute to their protective role against metabolic stress. The possible mechanism is that adiponectin plays an anti-inflammatory role through the blockade of NF-kappa B signaling, and it further inhibits the production of TNF-alfa. An adiponectin deficiency is an independent risk factor for endothelial dysfunction and cardiovascular complications. Also, omentin is negatively correlated with chronic inflammation, endothelial dysfunction, atherosclerosis, and calcification [53]. This adipokine is involved in lipid metabolism (through inhibiting oxLDL) and proper synthesis of nitric oxide through the inhibition of TNF-alpha-induced cyclooxygenase-2 (COX-2) expression, with further activation of the endothelial nitric oxide synthase (eNOS)/NO pathway [54]. Moreover, omentin is able to promote the anti-inflammatory M2 phenotype of macrophages during the differentiation of monocytes into macrophages, thus reducing the production of pro-inflammatory factors [16,19]. In this context, the increased ratios of adiponectin/leptin and omentin/leptin observed in our vegetarians may be related to a better metabolic profile in children who consume a well-planned vegetarian diet. It may protect against various chronic diseases in later life.

Other ratios regarding resistin and vaspin were without significant differences between the studied groups. Resistin plays an important role in inflammatory responses since the main sources of resistin secretion are macrophages [10]. Unexpectedly, in our study, serum resistin levels were positively correlated with the HMW/adiponectin ratio, and negatively with fat mass in vegetarians. No relations between resistin concentration and anthropometric parameters or other adipokine levels were observed in omnivores. The exact role of vaspin in the body is still a matter of debate. Some authors believe that vaspin has anti-inflammatory properties. We did not observe any associations of vaspin with body composition indicators in vegetarians; however, in omnivorous children, we found positive correlations between vaspin and LMI, as well between vaspin and the leptin/sOB-R ratio. Further studies are needed to clarify this issue.

In the analysis of correlations between adipokine ratios and dietary nutrient intakes, we observed that the percentage of energy from protein was significantly negatively related with the vaspin/resistin ratio, plant protein intake was positively related with the omentin/leptin ratio (*p* = 0.052), and dietary fiber with the adiponectin/resistin ratio in vegetarians. In the omnivores group, the percentage of energy from protein was negatively correlated with the adiponectin/leptin ratio and the omentin/leptin ratio, and positively with the vaspin/resistin ratio. The data regarding resistin, visfatin, and vaspin seems to be inconclusive and need further investigation.

There are only a few studies on the associations between a plant-based diet, adipokine secretion, and inflammation states. Kahleova et al. [35] reported that the vegetarian diet is more effective in increasing insulin sensitivity, reducing visceral fat volume, and improving serum adipokine levels than a conventional diet in patients with type 2 diabetes. The authors observed a decrease in leptin (by 35%) and resistin levels (19%), and an increase (by 19%) in total and HMW adiponectin levels in the studied patients after a 3-month dietary intervention. Also, McCarty et al. [55] suggested that, owing to their amino acid composition, plant proteins preferentially stimulate glucagon secretion, and as a consequence, they have favorable effects on adipocyte function, as increased adiponectin secretion, which directly exerts anti-inflammatory effects. In an experimental study on rats, Chen et al. [56], found that vegan diets have shown hypolipidemic effects and an increased expression of adiponectin and its receptor Adipo R1, which is involved in the reduction of gluconeogenesis via the AMP-activated receptor signaling pathway. It is difficult to explain whether changed levels of adipokines are directly associated with diet. Some authors suggested that polyphenols, vitamins, and fiber derived from plant-based foods are the main bioactive compounds that are associated with the anti-inflammatory and antioxidant properties [57,58,59]. It is not excluded that a lower dietary intake of total protein and animal protein, but a higher intake of plant protein and fiber observed in vegetarians may have a direct beneficial effect on body composition, fat tissue secretion, lipid profile, and glycemic control. One of the mechanisms that are likely to be responsible for improved glycemic control is increased insulin sensitivity in response to plant-based diets that partially replace of meats with soy products. Another potential mechanism is improved gastrointenstinal hormone response, especially of incretins [23]. Generally, it is known that too much protein (especially animal protein) in the diet is a predisposing factor for overweight/obesity in children. Also, high fiber consumption has been linked to reduced blood lipids, plaque formation, and cardiovascular risk.

The interpretation of serum adipokine concentrations with regard to their clinical use in pediatrics is hampered by a relative lack of reference values for children and adolescents. To our knowledge, our study is the first to determine and analyze the adipokine profile in children on a vegetarian diet. This represents a first attempt to study the interaction of diet and adipokines in the modulation of important metabolic processes. We think that future studies will provide information about implications of adipokine profile on public health research, and on public health practice. The beneficial adipokine profile in vegetarian children demonstrated in our study may indicate the benefits that are associated with the use of this diet in the context of the prevention of diseases underscored by inflammatory mechanisms. Some authors postulated strong association between the adipokine ratio (high leptin/adiponectin ratio) with a higher risk of type 2 diabetes, cardiovascular disease, or insulin sensitivity [60,61]. We suggest that ratios of other pro-inflammatory/anti-inflammatory adipokines may be useful in the prevention of dietary-related diseases as well as other inflammatory disorders as diagnostic tools in the future.

The results of our research are also an important contribution to the understanding of the pathomechanism of the impact of diet components on metabolic processes. Our previous study suggests that the leptin/adiponectin ratio may be a reliable parameter to assess the risk of obesity-related disorders in prepubertal children with simple obesity carrying different genotypes of the—11377C>G polymorphism in adiponectin gene [62]. The association between polimorphisms in other adipokine genes and pro-inflammatory/anti-inflammatory adipokine ratio may also reflect the relations between genetic variants, serum adipokine levels, and the inflammatory state. However, gaining more knowledge about these mechanisms requires future research on both children and adults, using different nutrition models.

Our study has several possible limitations. First, we analyzed relatively small samples, which reduced the power of our results. However, our data come from two groups of children who were similar in terms of age, weight, height, BMI, and body composition parameters. Second, we measured fat mass and percentage of fat mass, but did not examine the volume of subcutaneous and visceral fat, which could have provided a better tool for adipokine assessment than basic anthropometric parameters. Third, the obtained results were based on single measurements of the adipokines, and therefore do not reflect the long-term exposure of these hormones. However, we provided unique simultaneous evaluation of several adipokines with anti- and pro-inflammatory properties in children consuming vegetarian and omnivorous diets. Some of them such as visfatin, vaspin, and omentin, were examined in vegetarians for the first time. Fourth, we did not measure inflammatory markers, such as CRP, IL-6, or TNF-alpha, but the studied children are generally healthy without acute or chronic infections. Finally, the cross-sectional nature of our study does not permit for causality statements. However, we believe that our results may create basic knowledge for further studies evaluating the possible role of diet on the adipokine profile, inflammation, and metabolic abnormalities.

## 5. Conclusions

The results of our study suggest that the vegetarian diet affects the adipokine profile, and it may have a protective effect on inflammatory status. Higher ratios of anti- to pro-inflammatory adipokines, expressed as the adipectin/leptin and the omentin/leptin ratios, may suggest a better metabolic panel of adipokines in children consuming the vegetarian diet. Further studies with larger samples are necessary to elucidate the specific mechanisms linking dietary patterns and adipokine levels with metabolic and inflammatory markers.

## Figures and Tables

**Table 1:** Comparison of anthropometric parameters, and dietary energy and nutrient intakes between children on vegetarian and omnivorous diets.

	Vegetarians (*n* = 62)	Omnivores (*n* = 55)	*p*
Anthropometric parameters:			
Age (years) ^b^	6.0 (5–10)	6.5 (5–10)	0.313
Weight (kg) ^a^	22.1 ± 5.3	22.1 ± 6.4	0.975
Height (cm) ^a^	119.9 ± 12.1	120.2 ± 11.8	0.890
BMI (kg/m^2^) ^a^	15.3 ± 1.4	14.8 ± 1.5	0.078
Fat (%) ^a^	19.1 ± 5.5	21.2 ± 5.7	0.050
Fat mass (kg) ^a^	3.91 ± 1.58	4.36 ± 1.85	0.173
Lean mass (kg) ^a^	15.73 ± 3.07	16.01 ± 4.36	0.686
Fat/Lean	0.25 ± 0.09	0.28 ± 0.09	0.178
FMI (kg/m^2^) ^a^	2.67 ± 0.78	2.99 ± 0.86	0.044
LMI (kg/m^2^) ^a^	10.94 ± 1.33	11.09 ± 1.72	0.589
Dietary daily intake:			
Total energy (kcal) ^a^	1445 ± 490	1539 ± 397	0.303
Energy from protein (%) ^a^	11.7 ± 1.9	14.1 ± 4.0	<0.001
Energy from fat (%) ^a^	31.6 ± 5.0	32.8 ± 6.7	0.286
Energy from carbohydrates (%) ^a^	58.8 ± 5.3	53.1 ± 6.1	0.002
Protein (g) ^a^	41.7 ± 16.0	53.2 ± 19.4	0.002
Animal protein (g) ^b^	13.5 (8.5–20.3)	30.2 (22.5–38.4)	<0.001
Plant protein (g) ^a^	23.3 ± 10.4	19.3 ± 6.6	0.024
Fat (g)	52.8 ± 24.2	58.5 ± 23.2	0.234
Carbohydrates (g)	218.5 ± 68.7	213.9 ± 52.3	0.713
Fiber (g) ^a^	19.3 ± 8.5	15.6 ± 5.4	0.011

Data are presented as ^a^ mean values and standard deviation (SD), ^b^ median values and interquartile ranges (1Q–3Q); BMI: body mass index, FMI: fat mass index, LMI: lean mass index.

**Table 2:** Serum concentration of adipokines in vegetarian and omnivorous children.

	Vegetarians	Omnivores	*p*
Leptin/sOB-R ^b^	0.028 (0.015–0.048)	0.037 (0.026–0.061)	0.017
HMW/total adiponectin ^a^	0.586 ± 0.123	0.572 ± 0.191	0.639
Resistin (ng/mL) ^a^	4.14 ± 1.43	4.68 ± 1.56	0.051
Visfatin (ng/mL) ^b^	1.735 (0.772–2.669)	1.456 (0.910–2.901)	0.670
Omentin (ng/mL) ^b^	503.7 (287.0–996.6)	413.0 (269.5–1010.8)	0.330
Vaspin (ng/mL) ^b^	0.366 (0.258–0.647)	0.420 (0.330–0.606)	0.226

Data are presented as ^a^ mean values and standard deviation (SD) for normally distributed variables, or as ^b^ median values and interquartile ranges (1Q–3Q) for non-normally distributed variables; sOB-R—soluble leptin receptor, HMW—high molecular weight adiponectin.

**Table 3:** Correlation analysis between serum adipokine concentrations and anthropometric variables in children on vegetarian and omnivorous diets.

	Adipokines											
	Leptin/sOB-R		HMW/Adiponectin		Resistin		Visfatin		Omentin		Vaspin	
	*r*	*p*	*r*	*p*	*r*	*r*	*r*	*p*	*r*	*p*	*r*	*p*
**Vegetarians**												
Body weight	0.371	0.003	−0.148	0.252	−0.214	0.096	−0.178	0.167	−0.115	0.373	0.132	0.305
Height	0.400	0.001	−0.041	0.750	−0.132	0.306	−0.119	0.357	−0.050	0.698	0.146	0.258
BMI	0.174	0.177	−0.379	0.002	−0.199	0.121	−0.081	0.530	−0.224	0.080	0.019	0.886
Fat	0.475	0.000	−0.232	0.070	−0.305	0.016	−0.179	0.165	−0.057	0.659	0.047	0.718
Fat free	0.244	0.056	−0.075	0.561	−0.033	0.802	−0.022	0.864	0.018	0.890	0.182	0.157
Lean	0.060	0.617	0.213	0.289	−0.123	0.501	−0.117	0.334	−0.099	0.690	−0.213	0.191
FMI	0.242	0.058	−0.066	0.610	−0.026	0.838	−0.021	0.873	0.010	0.941	0.184	0.153
LMI	−0.291	0.022	−0.020	0.875	0.246	0.054	0.203	0.114	0.023	0.861	0.006	0.963
**Omnivores**												
Body weight	0.292	0.031	-0.107	0.435	−0.140	0.308	0.099	0.472	−0.049	0.741	0.161	0.270
Height	0.252	0.063	−0.207	0.129	−0.137	0.317	0.094	0.493	−0.076	0.602	0.125	0.392
BMI	0.191	0.162	0.070	0.609	−0.166	0.226	0.012	0.932	0.129	0.375	−0.006	0.967
Fat	0.221	0.126	0.006	0.970	0.063	0.666	0.032	0.827	−0.057	0.718	0.049	0.756
Fat free	0.106	0.470	−0.040	0.784	−0.139	0.342	0.058	0.691	−0.063	0.686	0.286	0.063
Lean	0.111	0.447	−0.039	0.788	−0.139	0.340	0.059	0.687	−0.057	0.718	0.297	0.053
FMI	0.130	0.372	0.088	0.549	0.085	0.562	0.047	0.748	0.001	0.993	−0.028	0.860
LMI	−0.107	0.462	0.214	0.141	−0.254	0.078	0.051	0.726	0.055	0.725	0.366	0.016

BMI—body mass index, FMI—fat mass index, LMI—lean mass index, sOB-R—soluble leptin receptor, HMW—high molecular weight adiponectin.

**Table 4:** Ratios of anti-inflammatory adipokines (adiponectin, omentin, vaspin) to pro-inflammatory adipokines (leptin, resistin) in children on vegetarian and omnivorous diets.

	Vegetarians	Omnivores	*p*
Adiponectin/Leptin	0.70 (0.37–0.93)	0.39 (0.28–0.74)	0.005
Adiponectin/Resistin	0.23 (0.16–0.31)	0.20 (0.14–0.25)	0.219
Omentin/Leptin	0.40 (0.23–0.83)	0.33 (0.15–0.48)	0.011
Omentin/Resistin	0.14 (0.08–0.26)	0.111 (0.07–0.19)	0.149
Vaspin/Leptin	0.26 (0.15–0.56)	0.24 (0.18–0.40)	0.792
Vaspin/Resistin	0.12 (0.07–0.16)	0.11 (0.08–0.14)	0.821

Data are presented as median values and interquartile ranges (1Q–3Q).

**Table 5:** Correlation analysis between ratios of anti- to pro-inflammatory adipokines and nutritional parameters in children on vegetarian and omnivorous diets.

	A/L Ratio		A/R Ratio		O/L Ratio		O/R Ratio		V/L Ratio		V/R Ratio	
	*r*	*p*	*r*	*p*	*r*	*p*	*r*	*p*	*r*	*p*	*r*	*p*
**Vegetarians**												
Energy intake	−0.085	0.546	−0.103	0.465	0.171	0.220	0.178	0.203	0.053	0.706	−0.004	0.979
Energy from protein	0.136	0.331	0.024	0.866	0.091	0.517	0.015	0.915	−0.207	0.137	−0.294	0.033
Energy from fat	−0.076	0.587	0.005	0.971	0.044	0.754	0.175	0.210	−0.051	0.714	0.114	0.417
Energy from carbohydrates	0.048	0.733	0.019	0.895	−0.035	0.804	−0.122	0.384	0.119	0.395	−0.015	0.914
Animal protein	0.003	0.983	0.045	0.746	0.140	0.317	0.194	0.163	−0.114	0.415	−0.092	0.512
Plant protein	0.072	0.610	−0.128	0.359	0.258	0.062	0.107	0.447	0.158	0.258	0.003	0.985
Fiber	0.005	0.972	−0.295	0.032	0.164	0.240	−0.055	0.694	0.179	0.201	0.023	0.868
**Omnivores**												
Energy intake	−0.084	0.577	−0.103	0,497	−0.023	0.889	−0.186	0.251	0.066	0.686	−0.210	0.193
Energy from protein	−0.335	0.023	−0.051	0.737	−0.341	0.032	−0.120	0.460	−0.011	0.946	0.328	0.039
Energy from fat	−0.023	0.881	−0.072	0.636	0.062	0.703	−0.099	0.544	0.107	0.513	−0.209	0.196
Energy from carbohydrates	0.215	0.151	0.054	0.720	0.067	0.682	0.094	0.563	−0.055	0.736	0.067	0.679
Animal protein	−0.289	0.052	−0.126	0.403	−0.255	0.112	−0.306	0.055	0.051	0.756	−0.065	0.691
Plant protein	0.180	0.232	0.040	0.793	0.198	0.222	0.108	0.508	0.121	0.458	0.049	0.764
Fiber	0.132	0.380	0.181	0.287	0.128	0.431	0.089	0.584	0.039	0.813	0.069	0.670

A/L—total adiponectin/leptin ratio, A/R—total adiponectin/resistin ratio, O/L—omentin/leptin ratio, O/R—omentin/resistin ratio, V/L—vaspin/leptin ratio, V/R—vaspin/resistin ratio.
